# “Crack, Reduce, and Implant”: A Safe Phaco Technique in a Case with Hard Brown Cataract

**DOI:** 10.1155/2019/9043417

**Published:** 2019-01-27

**Authors:** Ibrahim Toprak, Volkan Yaylali

**Affiliations:** Department of Ophthalmology, Faculty of Medicine, Pamukkale University, Denizli, Turkey

## Abstract

This report describes two maneuvers in different steps of phaco surgery in a case with hard cataract, which provide debulking of the central dense nucleus and prevents posterior capsule rupture (PCR). In the current case, clear corneal incisions were created, and anterior chamber was filled with ophthalmic viscosurgical device (OVD). Anterior capsule was punctured, and capsulorhexis was completed. Nucleus was cracked into two halves following vertical groove formation. Core nucleus was hollowed sideward bilaterally in the capsular bag. Nuclear halves were removed from capsular bag, and each one was pushed to one side on the iris plane. Capsular bag was inflated with OVD, and intraocular lens (IOL) was implanted. Nuclear halves were removed in confidence. The presented maneuvers initially reduce dense nucleus load in the safe zone and allow surgeon to use IOL as a barrier to protect floppy posterior capsule from early steps of the surgery.

## 1. Introduction

Phaco surgery in cases with hard brown cataract remains nightmare of eye surgeons and carries high risk of complications such as nucleus drop, posterior capsule rupture (PCR), corneal burn, and endothelial failure [1, 2].

Several manipulations have been described to prevent nucleus drop and vitreous prolapse when PCR develops during phaco surgery such as ophthalmic viscosurgical device (OVD) injection, intraocular lens (IOL) scaffolding, sheets' glide, and posterior assisted levitation [2–6]. However, none of the above-mentioned techniques are intended to prevent PCR. Recently, Parkash et al. [6] have presented an IOL scaffold technique to prevent PCR before complete or partial emulsification of the nucleus in morgagnian cataract.

The current study represents two facilitating maneuvers in different stages of phaco surgery in a case with hard brown cataract. This technique primarily aims at decreasing nucleus load in the safe zone and eliminating the risk for PCR.

## 2. Case Presentation

A 83-year-old woman presented with a visual acuity of light perception in the left eye for four years. The patient underwent detailed ophthalmological examinations including slit-lamp biomicroscopy, intraocular pressure measurement (applanation tonometry), dilated fundus examination (+90 D), and B-scan ocular ultrasonography. On initial examination, visual acuity was 0.2 (Snellen equivalent) in the right eye and light perception was in the left eye. Slit-lamp biomicroscopy revealed left hard brown cataract and right pseudophakia. Specular microscopy was within acceptable limits (cell density >2000 cells/mm^2^, hexagonality >50%) in the left eye. Intraocular pressure measurements were normal bilaterally. Right fundus examination showed macular atrophic changes. However, dense cataract did not allow a detailed fundoscopic evaluation in the left eye. B-scan ocular ultrasonography revealed no retinal detachment or intraocular mass.

**Figure 1:**
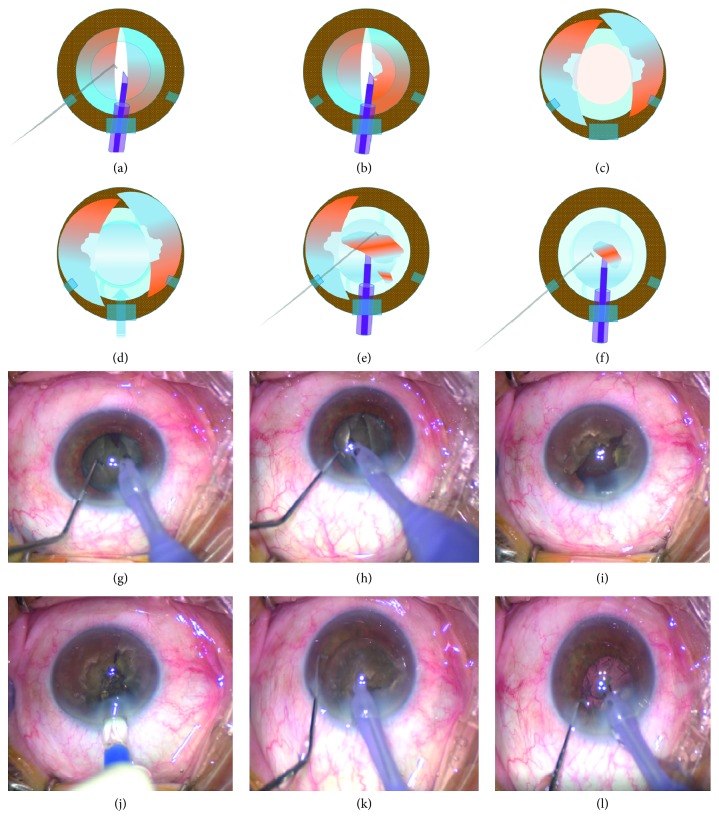
The illustration and intraoperative photographs present major steps of “*crack, reduce, and implant”* phaco technique. (a, g) Nucleus is cracked into two halves following vertical groove formation; (b, h) dense core of each halves is shaved sideward in the capsular bag to decrease nucleus load for further steps with low vacuum and phaco power; (c, i) with the assistance of ophthalmic viscosurgical devices (OVD), nuclear halves are prolapsed from the capsular bag to the iris plane; (d, j) anterior chamber and capsular bag are filled with OVD and a foldable intraocular lens (IOL) is implanted. Posterior capsule is now fully protected. (e, f, k, l) The nucleus is chopped into small pieces and removed far from the corneal endothelium with confidence, and the remaining cortical material is aspirated. Surgery is completed without complication.

Phaco surgery was performed under retrobulbar anesthesia due to poor patient compliance. A 2.75 mm superior clear corneal incision was created, and anterior lens capsule was stained with trypan blue dye. Central part of the anterior lens capsule was punctured with a cystotome following OVD injection into the anterior chamber (AC). A continuous curvilinear capsulorhexis was created. Two corneal side port incisions were made using a 20-gauge blade two clock hours away from the superior corneal incision. After hydrodissection, central vertical groove was formed, and nucleus was cracked into two halves (Figures 1(a) and 1(g)). Using the phaco needle, core nucleus (which is the rock-hard part of the lens) of the two halves was hollowed sideward in the capsular bag (using low vacuum (linear increment with an upper limit of 80 mmHg) and power (linear increment with an upper limit of 80% torsional amplitude) settings) (Figures 1(b) and 1(h)). A dispersive OVD was injected into the AC to protect corneal endothelium. The nuclear halves were removed from the capsular bag using the phaco tip, and each half was pushed to one side on the iris plane with assistance of OVD (Figures 1(c) and 1(i)). Capsular bag was inflated with OVD, and a foldable IOL was implanted (Figures 1(d) and 1(j)). Thus, posterior capsule moved backwards and got fully secured. Then, nuclear halves were chopped into small pieces and removed as far as possible from the corneal endothelium, which is also protected by dispersive viscoelastic (Figures 1(e), 1(f), 1(k), and 1(l)). After nucleus removal, the remaining cortical material and OVD were aspirated with bimanual irrigation/aspiration (I/A). Corneal incisions were hydrated, and intraocular antibiotic injection was performed.

Video 1 demonstrates the steps of the phaco surgery.

At first day postoperatively, biomicroscopic examination revealed normal findings except grade 2 corneal edema in the left eye. At 1-week control visit, corrected visual acuity was 0.7 (Snellen equivalent), cornea was clear, and minimal macular atrophic changes were present in her left eye. Postoperative control specular microscopy was planned at 1-month visit, whereas the patient was out of follow-up.

## 3. Discussion

Phaco surgery in cases with hard brown cataract is challenging, and PCR is not an uncommon complication in these cases. The necessity for more manipulation to remove sharp-edged nuclear fragments and floppy capsulozonular complex are the major risk factors for PCR in cases with hard cataract [1, 2, 6].

Most of the previous reports focused on management of PCR in phaco surgery to prevent dropping of nuclear remnants into vitreous. Kumar et al. [7] introduced IOL scaffold technique in which a three-piece foldable IOL blocks vitreous prolapse and nucleus drop. Narang and Agarwal [8] presented posterior-assisted levitation of sinking nucleus with IOL scaffold followed by glued IOL implantation in eyes with no sulcus support.

On the contrary, a number of methods have been presented for protecting posterior capsule from rupture in phaco surgery. In the study of Luo et al. [9], IOL is implanted in the capsular bag prior to the removal of the last nuclear fragment. A recent technique by Parkash et al. [6] has been introduced, which utilizes the IOL as a scaffold to prevent the PCR during phacoemulsification in morgagnian cataract. In this technique, the surgeon chops and removes a half of the nucleus prior to the IOL implantation. In the techniques of Luo et al. [9] and Parkash et al. [6], posterior capsule remains vulnerable during removal of the first nuclear pieces.

The presented maneuvers in our report provide two major advantages. First, sculpture of each nuclear core sideward in the capsular bag lowers the rock-hard nucleus load and provides advantage for further phaco steps. In this step, low vacuum and power setting is sufficient, and lens is too dense to thrust through. Therefore, risk for PCR is very low. After OVD injection into the capsular bag and IOL implantation, the posterior capsule retreats backward and gets fully safe. The IOL barrier also gives the surgeon the opportunity to get closer to the posterior plane while moving away from the corneal endothelium. Furthermore, a dispersive viscoelastic contributes endothelial protection. The surgeon removes both the nuclear halves (remaining) confidently with minimum maneuver, and removal of the hard nucleus outside the capsular bag minimizes the zonular stress. On the contrary, cortical removal does not cause a significant challenge even though the IOL is in the capsular bag because cortex in hard cataracts is generally liquefied or matured.

In conclusion, unlike previously introduced methods, the presented maneuvers in this case report initially reduce the hard nucleus load away from the corneal endothelium and utilize the IOL as a barrier to fully secure the floppy posterior capsule from beginning of the phaco surgery.

## Supplementary Materials

Supplementary Materials Video 1. This intraoperative video represents main maneuvers in the *“crack, reduce, and implant”* phaco technique in a case with dense cataract. Hard nucleus is cracked into two halves following groove formation, and central dense nucleus is hollowed sideward inside the capsular bag. Then, each piece is removed from the capsular bag to the iris plane with a phaco handpiece. Anterior chamber and capsular bag are inflated with viscoelastic, and a foldable intraocular lens is implanted, which protects the posterior capsule. Nuclear pieces are removed in the safe zone, and remaining cortex is aspirated. Surgery is completed successfully without any complication.
